# Dissolved Organic Carbon in Headwater Streams and Riparian Soil Organic Carbon along an Altitudinal Gradient in the Wuyi Mountains, China

**DOI:** 10.1371/journal.pone.0078973

**Published:** 2013-11-12

**Authors:** Wei Huang, William H. McDowell, Xiaoming Zou, Honghua Ruan, Jiashe Wang, Liguang Li

**Affiliations:** 1 Faculty of Forest Resources and Environmental Science, Nanjing Forestry University, Nanjing, Jiangsu, China; 2 Department of Natural Resources and the Environment, University of New Hampshire, Durham, New Hampshire, United States of America; 3 Institute for Tropical Ecosystem Studies, University of Puerto Rico, San Juan, Puerto Rico, United States of America; 4 Administrative Bureau of Wuyishan National Nature Reserve, Wuyishan, Fujian, China; 5 Soil and Water Science Department, University of Florida, Gainesville, Florida, United States of America; University of Maryland, United States of America

## Abstract

Stream water dissolved organic carbon (DOC) correlates positively with soil organic carbon (SOC) in many biomes. Does this relationship hold in a small geographic region when variations of temperature, precipitation and vegetation are driven by a significant altitudinal gradient? We examined the spatial connectivity between concentrations of DOC in headwater stream and contents of riparian SOC and water-soluble soil organic carbon (WSOC), riparian soil C:N ratio, and temperature in four vegetation types along an altitudinal gradient in the Wuyi Mountains, China. Our analyses showed that annual mean concentrations of headwater stream DOC were lower in alpine meadow (AM) than in subtropical evergreen broadleaf forest (EBF), coniferous forest (CF), and subalpine dwarf forest (SDF). Headwater stream DOC concentrations were negatively correlated with riparian SOC as well as WSOC contents, and were unrelated to riparian soil C:N ratio. Our findings suggest that DOC concentrations in headwater streams are affected by different factors at regional and local scales. The dilution effect of higher precipitation and adsorption of soil DOC to higher soil clay plus silt content at higher elevation may play an important role in causing lower DOC concentrations in AM stream of the Wuyi Mountains. Our results suggest that upscaling and downscaling of the drivers of DOC export from forested watersheds when exploring the response of carbon flux to climatic change or other drivers must done with caution.

## Introduction

Stream dissolved organic carbon (DOC) is suggested to be a major constituent of the global carbon cycle [1], [2]. At region to global scales, stream DOC concentrations and flux are largely governed by the quantity and quality of soil organic carbon (SOC). Soil organic carbon storage is an important driver of spatial variation in stream DOC fluxes [3]. At the global scale, DOC flux from watersheds is strongly related to soil C/N ratio [4], and similar relationships hold in some regions [5], [6]. Aitkenhead et al. [7] found that stream DOC concentrations could be predicted by SOC pools at three different scale-catchments with a range of soil types, land use and elevation. At a much smaller scale of investigation, Billett et al. [8] also showed that the spatial pattern of stream DOC concentrations was linked to changes of the SOC content in a small catchment (1.3 km^2^). At the stream-soil interface, the importance of the riparian zone in controlling carbon flow to the stream has been highlighted by many works [9], [10], [11]. For example, Dosskey and Bertsch [11] found that riparian soil contributed over 90% of DOC flux in a stream, even though they covered only 6% of the catchment area. Therefore, riparian SOC contents might have a first order control on stream DOC concentrations.

The delivery of SOC to streams can be understood by considering three fundamental processes: (1) partial decomposition of terrestrially fixed carbon producing DOC, (2) hydrologic transport of DOC from the terrestrial environment to streams, and (3) modification of both the quantity and composition of DOC during hydrologic transport [12], [13]. Thus, biotic and abiotic factors controlling DOC production, transport, and modification of quantity during transport, such as temperature [14], [15], precipitation and discharge [16], [17], soil moisture [15], [18], atmospheric nitrogen and SO_4_ deposition [19], [20], vegetative cover [21], [22], soil C:N ratio [4] and adsorption of DOC by soil [23], [24], are all likely to influence the relationship between stream DOC concentrations and SOC contents. For example, stream DOC concentration declined after the replacement of native deciduous forest by conifer plantations, suggesting that the change in vegetation resulted in a decline of organic matter decomposition and soil DOC production [21], [22]. Other analyses of data collected in streams have reported positive correlations between DOC and temperature, soil C:N ratio, and antecedent soil moisture [4], [8], [18].

Altitudinal gradients that span considerable variation of temperature, precipitation, vegetation and soil types over short geographic distances are well suited to study the relationship between stream DOC concentrations and SOC contents. However, few studies have shown a connection between stream DOC concentrations and SOC contents such along an altitudinal gradient. Aitkenhead et al. [7] reported that, for a given soil carbon pool, upland catchments with maximum altitude greater than 700 m have lower stream DOC concentrations than low land sites. Because precipitation was relatively constant among the studied watersheds (670.9 to 919.6 mm) [6], it was not thought to be a major driver of DOC concentrations.

The purpose of our study was to determine the relationship between stream DOC concentration and SOC content along an altitudinal gradient with considerable variation of temperature, precipitation, vegetation and soil types. The Wuyi Mountains have a clear vertical zonation of climate, vegetation and soil types in the subtropical monsoon region of China [25]. It provided an ideal site in which to investigate the connection between stream DOC concentrations and SOC contents. We hypothesized that: (1) DOC concentrations in headwater stream are driven by variation in riparian SOC content along the altitudinal gradient where variations of temperature, precipitation, and vegetation and soil types are large; (2) alternatively, stream DOC concentrations are more influenced by the variations of temperature, precipitation, and vegetation and soil types than by riparian SOC contents.

## Methods

### Site Descriptions and Experimental Design

The experimental sites were located in the Wuyi Mountain National Nature Reserve (27°33′–27°54′N, 117°27′–117°51′E), that occupies 565 km^2^ of forested land in the subtropical monsoon region of China [26]. The annual mean temperature is 15.0°C, with annual mean relative humidity of 83.5%, and 100 fog days per years. Annual precipitation reaches 2000 mm, most of which (60%) falls between later spring (April) and summer (July). There are four vegetation types along the altitudinal gradient from low to high elevation: subtropical evergreen broadleaf forest (EBF), coniferous forest (CF), subalpine dwarf forest (SDF) and alpine meadow (AM) (Table 1).

**Table 1:** Description of the four study sites located in the Wuyi Mountains, China.

Vegetationtype	Altitude (m)	Soil type	Dominant species	AMT (°C)	AMP (mm)	Litter mass(t hm^−2^ y^−1^)	Soil depth (cm)	Soil bulk density (g cm^−3^)	Soil pH	Soil silt plusclay (%)	Soil microbialbiomass(g kg^−1^)	Fine rootbiomass(kg m^−3^)
EBF	690	Humic Acrisols	*Castanopsis carlesii, Castanopsis eyrei*	18.5	1700	5.36a	0–10	0.87	4.54	31.17a	1.14a	3.06a
							10–25	0.89	4.82	36.79a	0.68a	0.75a
							25–40	0.96	4.94	40.12a	N/A	N/A
CF	1140	Humic Alisols	*Pinus taiwanensis*, *Oligostachyum oedogonatum*	14.5	2000	8.08b	0–10	0.64	4.64	38.07b	1.05a	7.01b
							10–25	0.77	4.79	37.79a	0.65a	1.99b
							25–40	0.87	4.88	45.24b	N/A	N/A
SDF	1750	Dystric Cambisols	*Symplocos paniculata*, *Stewartia sinensis*	11.2	2200	2.92c	0–10	0.61	4.59	41.93bc	1.46b	3.48a
							10–25	0.79	4.84	45.22b	0.71b	0.62a
							25–40	0.82	4.91	45.06b	N/A	N/A
AM	2060	Cambic Umbrisols	*Calamagrostis brachytrich*, *Miscanthus sinensis*, *Lycopodium clavatum*	9.7	3100	1.94d	0–10	0.54	4.86	47.85c	3.34c	9.73c
							10–25	0.65	5.13	51.33c	1.97c	1.21c
							25–40	0.80	5.22	60.77c	N/A	N/A

Note: AMT: annual mean temperature; AMP: annual mean precipitation. Datasets of annual mean temperature and annual mean precipitation are obtained from [25] and [26]. Datasets of soil bulk density and soil pH are obtained from [27]. Datasets of litter biomass, microbial biomass and fine root biomass are obtained from [28]. Different lowercase letters indicate significant differences among four study sites in the same soil layer.

**Figure 1:**
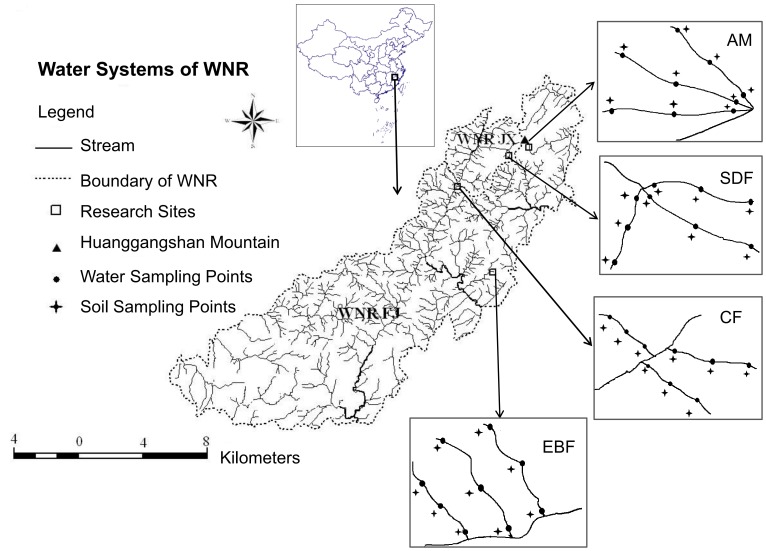
Map of headwater streams in the evergreen broadleaf forest (EBF), coniferous forest (CF), subalpine dwarf forest (SDF) and alpine meadow (AM) in the Wuyi Mountain National Nature Reserve of southeastern China (WNR, Wuyi Mountain National Nature Reserve; FJ, Fujian Province; JX, Jiangxi Province).

We selected four catchments in our study, one for each of the four vegetation types (Fig. 1) (Jiashe Wang issued the permission for each location of our study. He is the authority responsible for this national nature reserve). These catchments contain relatively pristine ecosystems that are currently not managed for agriculture or forestry. In each catchment, we selected three 1^st^ order streams (Stream orders were determined with the Strahler stream ordering system [29]). Within each 1^st^ order stream watershed, we identified three locations (the stream source, the stream middle − i.e. half way between the source and 2nd older stream, and the junction of the 1^st^ order with the 2^nd^ order stream). At each of these locations, we took stream water and their paired riparian soil samples (within 5 m from the stream bank). This experimental design resulted in 36 stream-water sampling points, 4 vegetation types × 3 streams × 3 locations; and its corresponding 36 riparian soil sampling points (Fig. 1).

### Field Sampling and Laboratory Analysis

We sampled stream water and soil every two months from June 2010 to April 2011. Over each 6-day of sampling period, we sampled water on days 1, 3 and 5, and soil samples on day 6. Water samples were collected in 100 ml HDPE bottles. Water samples from three locations of each stream were mixed in field. All water samples were field-filtered through pre-combusted glass fiber filters (0.7 µm Whatman GF/F), kept in a cooler during transportation to the laboratory, and stored at 4°C until analysis within 72 h. Three soil layers were sampled (i.e. 0–10 cm, 10–25 cm and 25–40 cm) from all plots using a 2 cm diameter soil corer.

Soil samples from three locations of each stream riparian zone were composited, sieved (<2 mm) to remove soil macroorganisms, rocks and fine roots, placed in plastic bags and transported in a cooler, thoroughly mixed and then divided into two parts. One part was kept in the refrigerator at 4°C before analysis for water-soluble soil organic carbon (WSOC) content, and the other part was air-dried and sieved (0.25 mm) for SOC and total nitrogen (TN) analysis.

Temperature of stream water and soil at 5 cm depth was measured using an electronic thermometer. Soil moisture was gravimetrically determined using the difference in weight before and after drying a soil sample (24 hours, 105°C). The particle-size was determined by wet sieving and sedimentation using the pipette sampling technique.

Stream DOC (measured as non-purgeable organic carbon) was analyzed via high temperature combustion on a Shimadzu TOC/TN–V_CPN_ analyzer (Shimadzu Corp. Japan).

Water-soluble soil organic carbon was extracted from 20 g of field moist soil samples (<2 mm sieved) with distilled water (1∶2 soil-water ratio) [30], [31]. The suspension was shaken for 30 min, centrifuged for 20 min at 4000×g, and filtered through pre-combusted glass fiber filters (0.7 µm Whatman GF/F). Organic carbon concentration of the extracts was determined with a Shimadzu TOC-V_CPN_ analyzer (measured as the difference between total carbon and inorganic carbon). The WSOC content of soil sample was expressed on the basis of equivalent oven dry weight.

Soil organic carbon and TN content were determined by a C/N/S-Analyzer (Vario EL III, Elementar, Germany). The method detection limits for SOC and TN are 0.03 mg C and 0.03 mg N, respectively.

### Statistical Analysis

All statistical analyses were performed using SPSS 17.0. Due to the small replications (n = 3), we employed Kruskal-Wallis test to determine if there were statistically significant differences in stream DOC concentrations, riparian SOC contents, riparian WSOC contents, riparian soil C:N ratio and temperature among vegetation types. Linear regression models were used to evaluate relationships between stream DOC concentrations and temperature, riparian SOC contents, riparian WSOC contents, riparian soil C:N ratio, as well as the relationship between riparian WSOC contents and SOC contents. When we performed liner regression, data from all four sites were analyzed together. Before performing linear regression, we used Durbin-Watson, histogram of errors and scatter diagram of errors to test the validity of these linear regressions. Significance was defined as p<0.05.

## Results

**Figure 2:**
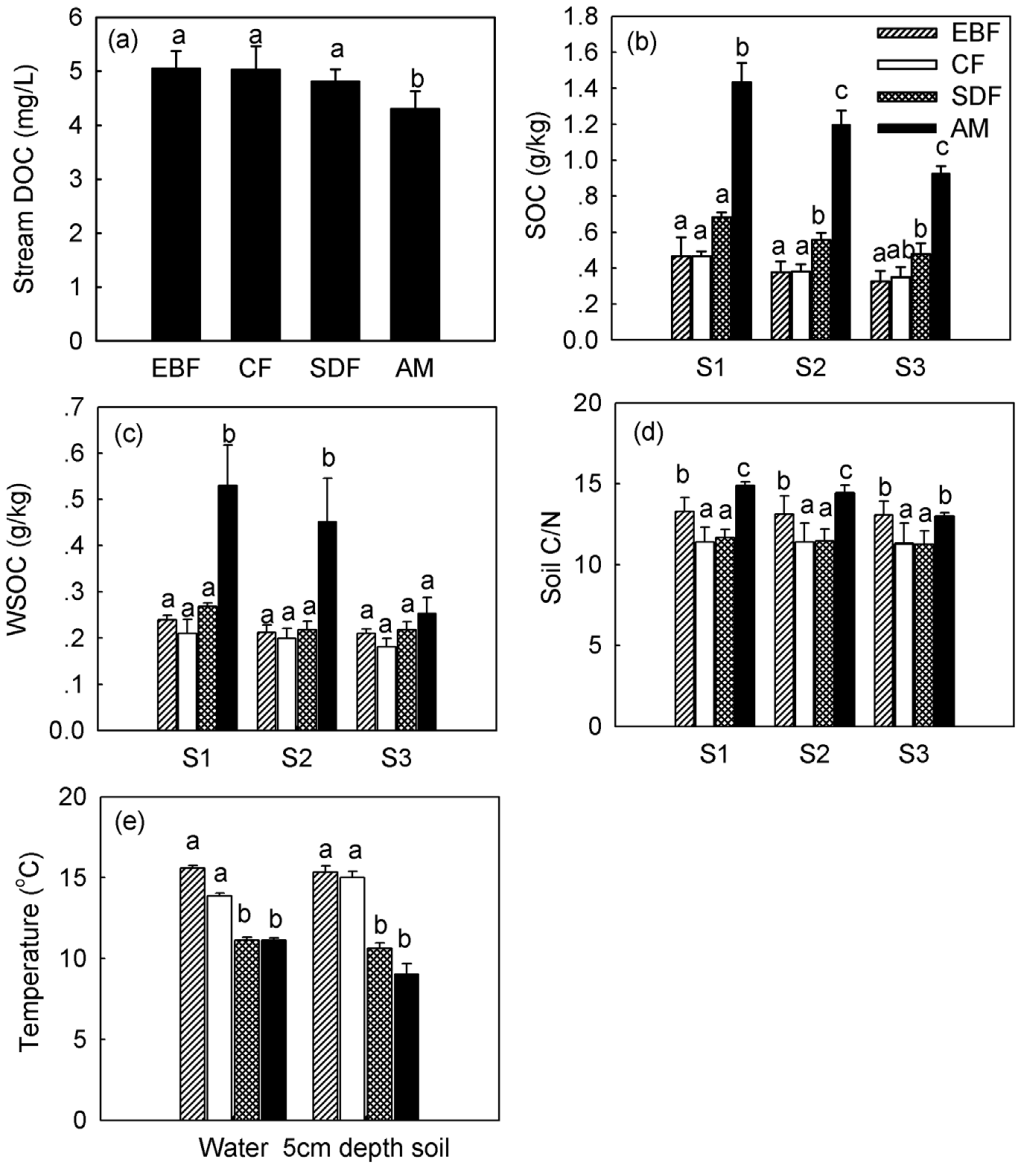
Annual mean values in (a) headwater stream dissolved organic carbon (DOC) concentrations, (b) riparian soil organic carbon (SOC) contents, (c) riparian water-soluble organic carbon (WSOC) contents, (d) riparian soil C:N ratio, and (e) temperature (mean±SD, n = 3) along an altitudinal gradient in the Wuyi Mountains of China (S1∶0–10 cm soil layer; S2∶10–25 cm soil layer; S3∶25–40 cm soil layer; EBF: evergreen broadleaf forest; CF: coniferous forest; SDF: subalpine dwarf forest; AM: alpine meadow; DOC: dissolved organic carbon; SOC: soil organic carbon; WSOC: water-soluble soil organic carbon). Significant differences between the means are marked with different letters.

Annual mean concentrations of stream DOC were lower in alpine meadow (AM) than in subtropical evergreen broadleaf forest (EBF), coniferous forest (CF), and subalpine dwarf forest (SDF), exhibiting a decreasing pattern with increasing altitude (Fig. 2a, Table 2). In contrast, annual mean contents of riparian SOC were higher in AM than in other vegetation types in all three soil layers, which showed an increasing trend with altitude (Fig. 2b, Table 2). Annual mean contents of riparian WSOC were also higher in AM than in other vegetation in 0–10 cm and 10–25 cm soil layers (Fig. 2c, Table 2). Riparian soil C:N ratio was significantly higher from AM and EBF than for samples from SDF and CF in all three soil layers (Fig. 2d, Table 2), but did not show an altitudinal trend. Annual mean stream water temperature and riparian soil temperature at 5 cm depth were higher in the EBF and CF catchments than in SDF and AM catchments (Fig. 2e).

**Table 2:** Annual mean data of stream DOC concentration and SOC character (mean±SD, n = 3).

Vegetation type	Stream DOC concentration (mg L^−1^)	SOC (g kg^−1^)			WSOC (g kg^−1^)			Soil C/N		
		0–10 cm	10–25 cm	25–40 cm	0–10 cm	0–25 cm	25–40 cm	0–10 cm	0–25 cm	25–40 cm
EBF	5.06±0.32a	0.47±0.11a	0.38±0.06a	0.33±0.06a	0.24±0.01a	0.21±0.02a	0.21±0.01a	13.30±0.85b	13.11±1.14b	13.09±0.83b
CF	5.03±0.44a	0.47±0.03a	0.38±0.04a	0.35±0.06a	0.21±0.03a	0.20±0.02a	0.18±0.02a	11.40±0.92a	11.40±1.16a	11.28±1.28a
SDF	4.80±0.23a	0.68±0.11a	0.56±0.08b	0.48±0.04b	0.27±0.01a	0.22±0.02a	0.22±0.02a	11.70±0.50a	11.45±0.76a	11.25±0.83a
AM	4.30±0.32b	1.43±0.19b	1.20±0.25c	0.93±0.13c	0.53±0.08b	0.45±0.09b	0.25±0.03a	14.88±0.23c	14.43±0.50c	12.99±0.21b

Different lowercase letters indicate significant differences among four study sites.

**Figure 3:**
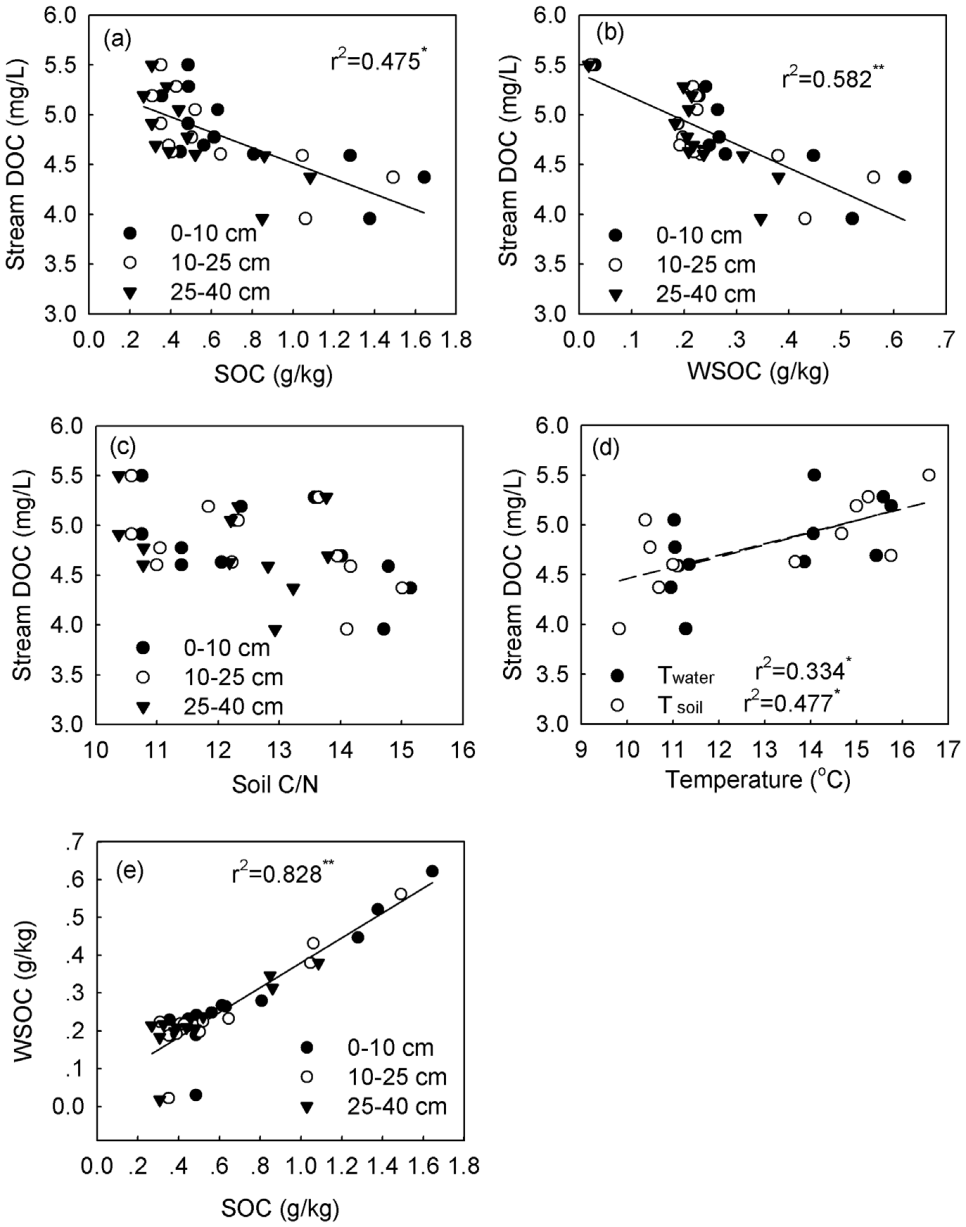
Correlations between annual mean headwater stream dissolved organic carbon (DOC) concentrations and (a) riparian soil organic carbon (SOC) contents, (b) riparian water-soluble organic carbon (WSOC) contents, (c) soil C:N ratio, (d) temperature, and (e) the link between riparian WSOC contents and riparian SOC contents along an altitudinal gradient in the Wuyi Mountains of China (T_water_: water temperature; T_soil_: soil temperature; n = 12. ^*^and ^**^indicated significant levels at 0.05 and 0.01 level, respectively).

Headwater stream DOC concentrations were correlated negatively with both riparian SOC and WSOC contents across all four vegetation types along the altitudinal gradient (Fig. 3a, b). There was no significant correlation between riparian soil C:N ratio and headwater stream DOC concentrations (Fig. 3c). Positive correlations were found between headwater stream DOC concentrations and stream temperature or riparian soil temperature at 5 cm depth (Fig. 3d). Contents of riparian WSOC were positively correlated with contents of riparian SOC (Fig. 3e).

## Discussion

Contrary to patterns observed in past studies, the DOC concentration of twelve headwater streams in the Wuyi Mountains of China decreased as a function of SOC content, rather than showing the increase in DOC that would be expected from earlier work such as Aitkenhead et al. [7]. Although we studied only riparian SOC and WSOC content in our attempt to link soil and stream DOC concentration, previous work in the Wuyi Mountains also shows very similar overall trends for non-riparian soils. Xu et al. [32] found SOC and its labile fractions, i.e. readily oxidizable carbon, WSOC and microbial biomass carbon increased with altitude. Thus stream DOC concentrations were negatively related to many measures of soil carbon and carbon fractions, and are also unrelated to soil C/N ratio, which was suggested to drive spatial variability in stream DOC flux at the global scale [4].

There are several possible explanations for the negative correlation between stream DOC and soil organic carbon that are opposite to those observed in other studies. We can rule out spatial variability in soils, as we sampled soils extensively in the riparian zones that are thought to preferentially supply streams with DOC [33], [34], and past work at the site shows that watershed soils show strong patterns in carbon with elevation identical to those we have quantified in riparian soils [32]. It also seems unlikely that our stream sampling, which occurred multiple times throughout the year (a total of 6 individual samples), would be insufficient to capture the basic pattern in concentrations of stream DOC as a function of watershed elevation. Our sampling was not sufficient to capture all the hydrologic extremes at a sampling site, but because it was consistent across elevation, with all elevations sampled on each sampling date, the patterns in stream chemistry should be relatively insensitive to hydrologic variation.

There are three fundamental processes that control the transport of carbon from soils to streams: DOC production in soil, hydrologic transport of DOC from soil to stream, and modification of DOC during transport [12]. In soils, DOC production depends on environmental factors such as climate (temperature, precipitation), soil properties, and vegetation types [35], [36]. The hydrologic transportation of DOC is influenced by precipitation [37]. And the modification of quantity of DOC during transport appears to be controlled by soil properties [23], [24]. In our study, the negative relationship between stream DOC concentrations and riparian SOC contents may be due to the large changes in precipitation across our study sites (from 1700 mm in EBF to 3100 mm in AM) and differences in soil clay plus silt contents among soils from the four different vegetation types.

Increasing precipitation could lead to a dilution effect on DOC in soil and stream water, and consequently to decrease DOC concentrations in streams [38]. The increased precipitation with altitude [26] suggests that DOC concentrations in riparian soil and stream would nearly be diluted by a factor of 2 at the highest elevation catchment when compared with the lowest.

The capacity of catchment soils to adsorb DOC is an important driver of stream DOC concentrations [23], [24]. Adsorption capacities of soil typically vary as a function of their clay contents [24]. AM soil had higher clay plus silt contents than other vegetation types in all three soil layers. This higher clay plus silt content in AM soil might lead to higher adsorption capacity than in other vegetation types. This might result in lower export of soil DOC in AM than in other vegetation types for a given soil DOC concentration at the same temperature.

Armstrong et al. [39] suggested that vegetation controlled the DOC concentrations of drain water through affecting microbial assemblages, root exudates and litter quantity and quality associated with different plant functional types. Our data show that DOC production in soils is highest in the AM (with its high WSOC), but that loss of DOC along hydrologic flow paths is sufficiently large to lead to the lowest stream DOC concentrations. Fine root and microbial biomass in AM were also higher than those in other three vegetation types. Although the highest litter biomass occurred in CF, litter in CF was slow to decompose due to the large amount of waxes, resins and lignin contents [40]. These fine root and microbial biomass, and the quality and quantity of litter in the Wuyi Mountains reflect, in part, that soil DOC production varies with vegetation types. But survey of our study showed that stream DOC concentrations and WSOC contents presented oppositely spatial pattern along the elevation gradient. Thus we conclude that soil DOC production, which is associated with vegetation, isn’t the most important driver of stream DOC concentrations in the Wuyi Mountains.

Vegetation may also affect the transport of soil DOC by affecting the chemical composition of DOC, soil solution pH, and soil physical structure that influence soil water infiltration [41], [42], [43]. The hot-water extractable organic matter from CF soils of Wuyi Mountains contains more highly condensed aromatic and hetero cyclic compounds than other three vegetation types, leading to being preferentially retained in the upper soil layer [27]. But DOC concentration in CF stream wasn’t significantly lower than the other three vegetation types. Thus, the spatial pattern of stream DOC concentrations in our study is not likely to be controlled by the influence of vegetation on soil DOC transportation by affecting the chemical composition of DOC. Hardine et al [43] demonstrated that changes in soil solution pH could affect the adsorption efficiency of DOC in minerals. The differences in soil pH across four different vegetation types were rather small. It is unlikely that these differences in soil pH could explain the observed altitudinal pattern in stream DOC concentrations. Vegetation can also affect soil physical properties thus soil water infiltration rate, through producing root tunnels and influencing soil microbial and faunal community structure and their activities [42]. Unfortunately, we do not have data on either water infiltration rates or the biomass of plant large roots and soil fauna to evaluate vegetation influences on soil DOC adsorption and transport.

Temperature is known to stimulate biological activity and decomposition rates, affecting the production of DOC from soil organic matter [44], [45]. However, our data showed both SOC and WSOC were highest where temperature and stream DOC concentrations were lowest, in the high elevation streams. Thus, we suggest that temperature isn’t the primary driver of the negative association between stream DOC concentration and SOC along an altitudinal gradient in the Wuyi Mountains.

## Conclusion

We found a negative relationship between stream DOC concentrations and SOC contents under four different vegetation types along an altitudinal gradient in the Wuyi Mountains of China. The highest stream DOC concentration was in EBF and lowest in AM. Variations in precipitation and soil clay plus silt content in AM appear to be the dominant control on spatial variation of stream DOC concentrations along the altitudinal gradient in Wuyi Mountains. Our data from the Wuyi Mountains show that the drivers of spatial variability in stream DOC concentrations along a mountain transect can be directly opposite to those obtained from regional or global analyses. This strong relationship between standing condition and DOC export reflects some of the inherent difficulties in extrapolating the results from regional or global models of stream DOC to the steep environmental gradients occurring in mountainous terrain. Under such a situation, extrapolation of the drivers inferred from global comparisons to individual sites must be done with great caution. Furthermore, our results suggest that predicting changes in DOC concentrations in response to changing climate at a given site must be also done with considerable caution, as it is unclear whether changes in soil carbon would be expected to increase or decrease the delivery of DOC to streams from watershed soils. Further research effort is required to establish multi-factors regression model, which include physical (climate and soil) and biotic (chemistry and quantity of organic inputs) variables, to predict stream DOC concentration.
